# Gut microbiota and hypertension: a bibliometric analysis of recent research (2014–2023)

**DOI:** 10.3389/fnut.2023.1253803

**Published:** 2023-10-12

**Authors:** Yang Jiao, Wenxing Li, Qianyi Zhang, Qianfeng Jiang

**Affiliations:** ^1^Department of Cardiology, Zunyi First People's Hospital, The Third Affiliated Hospital of Zunyi Medical University, Zunyi, Guizhou, China; ^2^Zunyi Medical University, Zunyi, Guizhou, China; ^3^Department of Cardiology, Guizhou Aerospace Hospital, Zunyi, Guizhou, China

**Keywords:** gut microbiota, hypertension, short-chain fatty acids, salt-sensitive hypertension, bibliometrics

## Abstract

**Background:**

Cardiovascular diseases persist as the primary cause of mortality in the global population. Hypertension (HTN) is widely recognized as one of the most crucial risk factors contributing to severe cardiovascular conditions. In recent years, a growing body of research has highlighted the therapeutic potential of gut microbiota (GM) in addressing cardiovascular diseases, particularly HTN. Consequently, unraveling and synthesizing the connections between GM and HTN, key research domains, and the underlying interaction mechanisms have grown increasingly vital.

**Methods:**

We retrieved articles related to GM and HTN from 2014 to 2023 using Web of Science. Bibliometric tools employed in this analysis include CiteSpace and VOSviewer.

**Result:**

From 2014 to 2023, we identified 1,730 related articles. These articles involved 88 countries (regions) and 9,573 authors. The articles were published in 593 journals, with 1000 references exhibiting co-occurrence more than 10 times. The number of studies in this field has been increasing, indicating that it remains a research hotspot. We expect this field to continue gaining attention in the future. China leads in the number of published articles, while the United States boasts the most extensive international collaborations, signifying its continued prominence as a research hub in this domain. Tain You-Lin, Hsu Chien-Ning, Raizada Mohan K, and Yang Tao are among the authors with the highest publication volume. Publications in this field are frequently found in nutrition, cardiovascular, and molecular biology journals. The most frequently occurring keywords include metabolic syndrome, cardiovascular disease, inflammation, short-chain fatty acids, trimethylamine N-oxide, chronic kidney disease, heart failure, and high-salt diet.

**Conclusion:**

The relationship between GM and HTN is presently one of the most active research areas. By employing bibliometric tools, we analyzed critical and innovative articles in this field to provide an objective summary of the primary research directions, such as the relationship between GM and HTN, GM metabolites, high-salt diet, the developmental origins of health and disease, obstructive sleep apnea-Induced hypertension and antihypertensive peptide. Our analysis aims to offer researchers insights into hotspots and emerging trends in the field of GM and HTN for future research reference.

## 1. Introduction

Cardiovascular disease poses a significant global health challenge and remains a leading cause of death. The prevalence of cardiovascular disease continues to rise, and hypertension (HTN) is among the primary risk factors for severe cardiovascular conditions (1, 2). Consequently, finding novel approaches to manage blood pressure is essential. The human body houses approximately 100 trillion microorganisms, with the majority residing in the intestines, forming the human gut microbiota (GM). This GM plays a role in regulating multiple bodily systems, including the cardiovascular, respiratory, and digestive systems. Recent studies have demonstrated that cardiovascular diseases, such as HTN, heart failure, myocardial infarction, and atrial fibrillation, are closely associated with the GM. Among the research exploring the connections between cardiovascular diseases and the GM, HTN has been an early and prominent concern. In 2015, Yang et al. established a correlation between GM and HTN (3). In 2017, Li et al. induced HTN in normal rats by transplanting GM from hypertensive patients (4). GM can metabolize dietary fiber into short-chain fatty acids (SCFAs). Francine et al.'s team provided hypertensive rats with dietary fiber or SCFAs, achieving a reduction in systolic and diastolic blood pressure (5). Presently, nutritionists advocate for increased dietary fiber consumption to lower the risk of cardiovascular disease (6). Consequently, conducting in-depth investigations into the association between GM and HTN bears significant implications for the treatment and prevention of cardiovascular diseases.

Bibliometrics, unlike traditional review articles, employ mathematical and statistical techniques to analyze texts and identify current trends and pressing issues in a specific research field (7). In recent times, researchers have carried out bibliometric analyses on GM in relation to atherosclerosis, heart failure, and other aspects (8, 9). However, as HTN poses one of the most substantial threats to cardiovascular health, it has yet to be explored through a corresponding bibliometric analysis. Therefore, there is an immediate demand for a bibliometric study to uncover the latest viewpoints and trending developments concerning the role of GM in HTN.

## 2. Materials and methods

### 2.1. Data sources and search strategy

**Figure 1:**
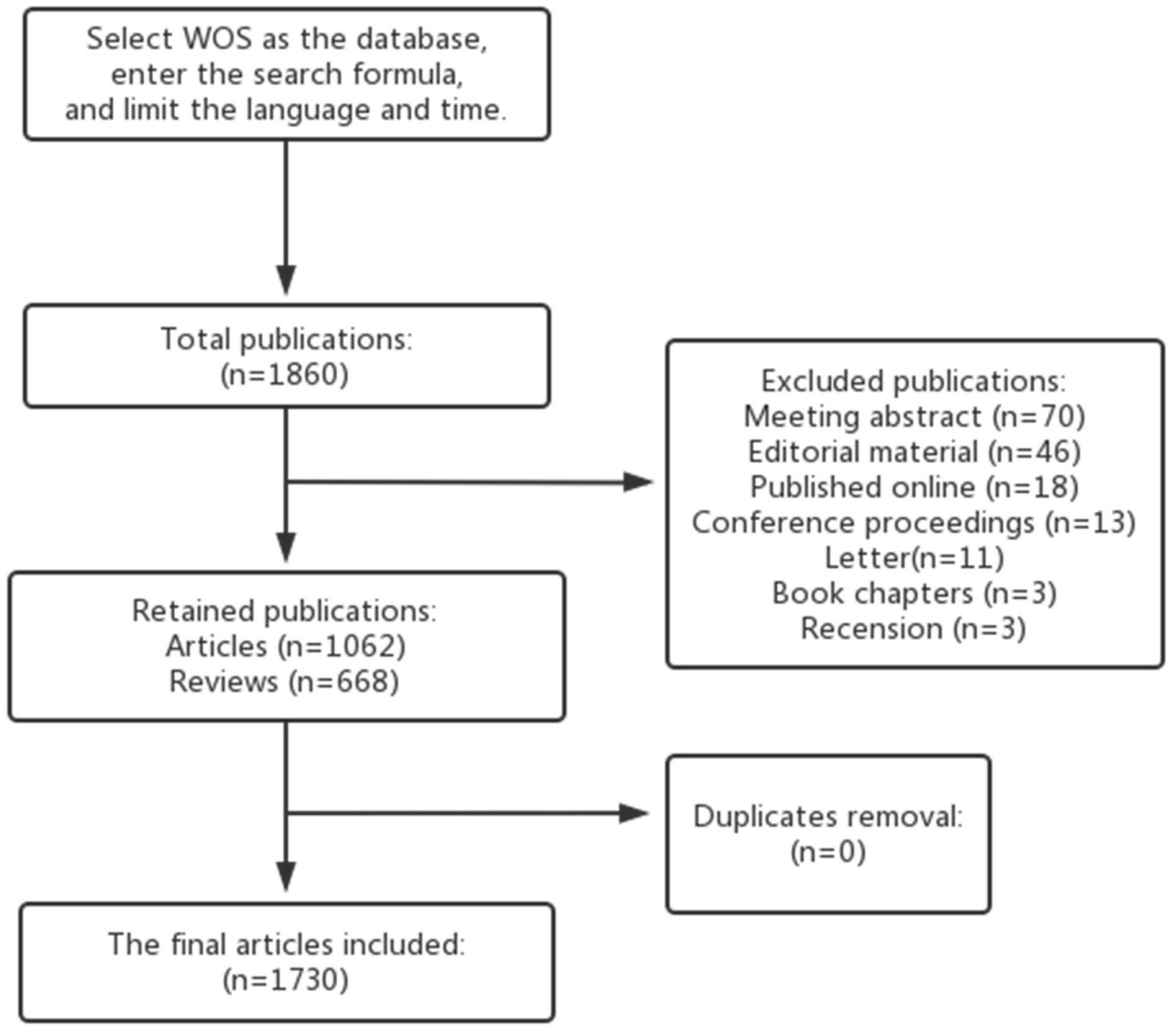
Flowchart for including and excluding publications.

A broad range of scholars concur that the Web of Science (WOS) is a reliable database ideally suited for bibliometric research. We extracted articles related to GM and HTN from the WOS database. To minimize search bias caused by database updates, we employed the following search query: TS = [(Microbiome* OR Microflora* OR Microbiota* OR Flora OR “Microbial Community” OR Bacteria) AND (Gastrointestinal OR Gut OR Gastric* OR Intestinal)] AND TS = (Hypertension OR “High Blood Pressure*”). The search period spanned from January 1, 2014, to February 28, 2023, to further reduce bias. We limited our search to articles and reviews in English, with Figure 1 illustrating the specific flow chart.

### 2.2. Data analysis

We use VOSviewer and CiteSpace as bibliometric analysis tools to conduct a quantitative analysis of the countries, authors, journals, keywords, and references associated with GM and HTN research. VOSviewer is utilized to create visual network representations of countries, authors, and journals, as it effectively displays extensive bibliometric maps in more comprehensible images (10). Simultaneously, we use CiteSpace to analyze keywords and references in this domain, as the timelines, burst, and clustering analyses generated by CiteSpace enable a more in-depth analysis of the hotspots and frontiers in GM and HTN research, offering valuable insights for future studies.

## 3. Results

### 3.1. Analysis of growth trends of annual publications

**Figure 2:**
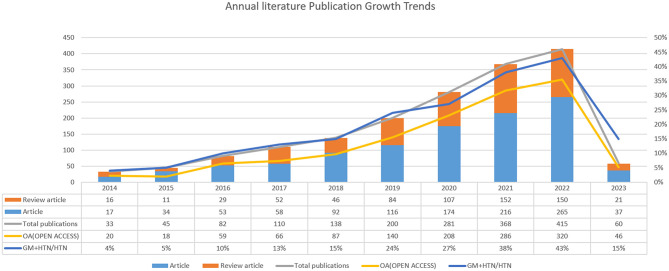
Annual literature publication growth trends.

An annual publication trend graph offers insight into the evolution and growth of a particular research field. In our study, we collated a total of 1,730 pertinent articles, comprising 1,062 research articles and 668 review articles. Out of these, 1,250 (72%) were open access, facilitating free knowledge dissemination, thereby promoting scientific progress in this field. As depicted in Figure 2, the research interest in this field has shown a consistent and rapid upward trajectory. Between 2014 and 2016, less than 100 articles were published annually. However, the publication rate markedly increased from 2017 to 2022, starting with 110 articles in 2017 and exhibiting an approximate annual addition of 80 articles thereafter. The year 2022 saw the publication of over 400 articles. Moreover, the proportion of GM+HTN research in relation to the broader field of hypertension-related research rose from 4% in 2014 to 43% in 2022. These trends suggest a continued and growing interest in the intersection of GM and HTN, indicating that this area will remain a focal point for future research.

### 3.2. Analysis of countries (regions)

**Figure 3:**
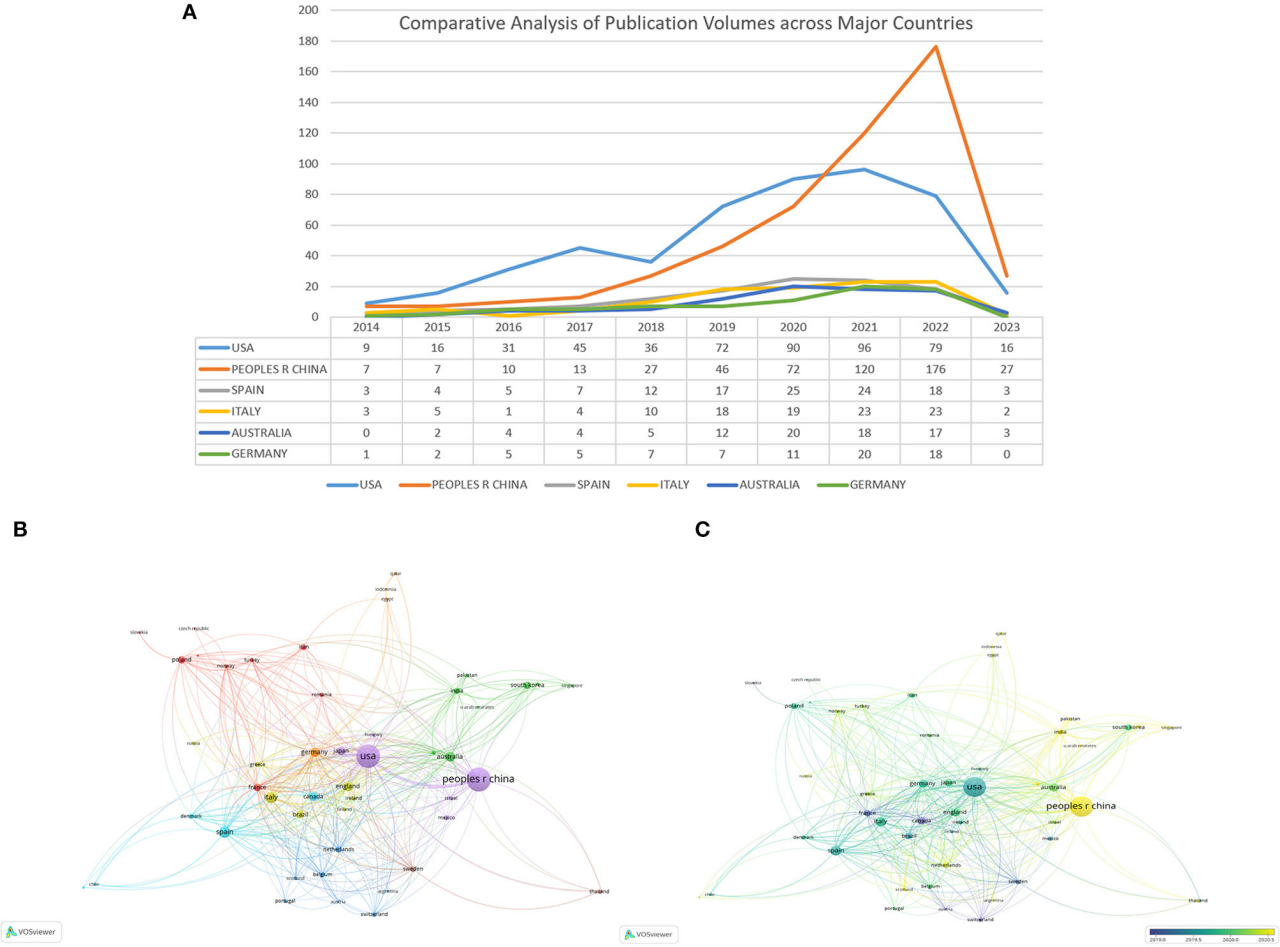
**(A)** Comparative analysis of publication volumes across major countries. **(B)** Collaboration network map among countries/regions using VOSviewer. **(C)** Timeline graphs displaying interactions among 88 countries/districts using VOSviewer the node size signifies the quantity of articles. Node color depicts the research topic, while lines connecting nodes indicate cooperative relationships between countries. A wider line between two nodes represents greater collaboration between the countries involved. Additionally, a node connected to numerous other nodes demonstrates its recognition and influence across multiple countries.

Among 88 nations, China stands as the leading contributor in terms of article publication, boasting 500 articles and a total citation count of 10,953 (Table 1). However, China's relatively low Centrality and average citation numbers suggest a need for more original research. The United States follows closely with 487 published articles and a substantial 20,308 citations. Among the top ten publishing countries, Germany holds the record for the highest average number of citations, at 52.4. Figure 3A showcases the yearly publication volumes across countries, where China and the United States outshine others in terms of output. Prior to 2020, the United States consistently led in publication volume. However, in 2021, China experienced a significant surge in output, surpassing the United States. The publication volumes of other countries have shown a relatively steady trend over the past decade. To further explore the interconnections between different countries, we utilized VOSviewer for a visual analysis of collaboration relationships and timelines. Figure 3B elucidates the collaborative ties between countries. Notably, the United States has forged the most extensive collaborative relationships, positioning itself at the heart of this research field. It shares its highest level of collaboration with China. Figure 3C reveals the average research initiation time in this field for different countries. Nations like Canada, France, Sweden, and Switzerland embarked on research in this area before 2019. The United States, Germany, Italy, and others began their involvement around mid-2019. Meanwhile, China, India, the Netherlands, and others initiated their research endeavors in this field around 2020.

**Table 1:** Top 10 countries/regions with the highest number of published articles.

**Rank**	**Country**	**Documents**	**Citations**	**Average number of citations**	**Centrality**	**Year (Start of Research)**
1	Peoples R China	500	10953	21.9	0.06	2014
2	USA	487	20308	41.7	0.47	2014
3	Spain	118	5050	42.8	0.09	2014
4	Italy	108	4632	42.9	0.14	2014
5	Australia	85	3080	36.2	0.17	2015
6	Germany	78	4084	52.4	0.25	2014
7	England	75	3598	48.0	0.10	2014
8	Canada	69	2458	35.6	0.03	2014
9	Japan	63	2002	31.8	0	2016
10	Brazil	58	1880	32.4	0.01	2014

### 3.3. Analysis of authors

**Figure 4:**
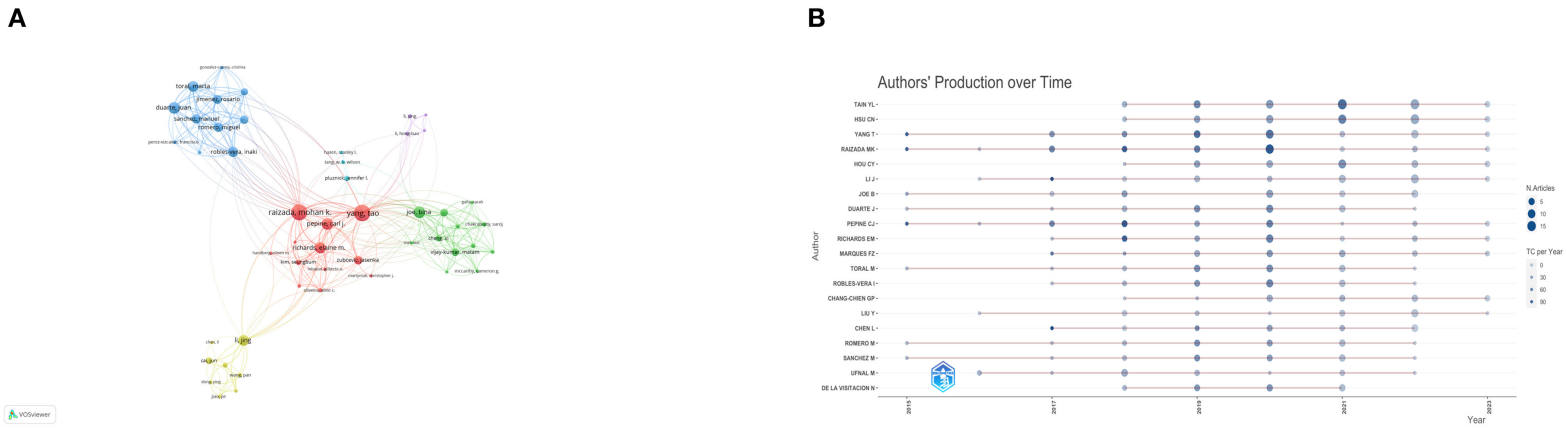
**(A)** Collaboration network map among authors using VOSviewer. **(B)** Authors' production over time.

To identify leading contributors in the field, we performed a visual analysis of authors (Table 2). We used Price's law, a rule of thumb which suggests that half of the publications in a specific field are made by the square root of all contributors, to designate core authors as those who have published more than 19 articles. Among these, Tain You-Lin and Hsu Chien-Ning lead the pack with the highest publication counts of 51 and 42 articles, respectively. Yang Tao, Raizada Mohan K, and Hou Chih-yao have outstanding citation percentages of 98, 90.38, and 100%, respectively, with their work falling within the 85th, 71st, and 76th citation percentiles. We then employed VOSviewer for a visual representation of author clusters, focusing on authors who have published more than 5 articles. Authors publishing on similar research topics were grouped into same-color clusters (Figure 4A). Within the red cluster, Raizada Mohan K and Yang Tao show extensive collaborations with other authors and stand out as some of the most influential contributors. In the blue cluster, Robles-vera, Inaki, and Romero, Miguel are also recognized as influential authors. To examine the recent publication patterns of these authors, we analyzed the publication volumes of the top 20 authors over the past decade (Figure 4B). All these authors have published articles within the last 5 years, with the peak of publication volume appearing between 2018 and 2021.

**Table 2:** Top 10 Authors with the highest number of published articles.

**Rank**	**Author**	**Documents**	**Citations**	**CICN (2018–2022)**	**H-Index**	**% Documents cited (2018–2022)**	**Citation percentile**
1	Tain, You-lin	51	963	1.06	41	87.27%	65th
2	Hsu, Chien-ning	42	747	0.98	32	77.14%	68th
3	Yang, Tao	34	2335	3.81	25	95.00%	85th
4	Raizada, Mohan K.	33	2775	5.63	72	90.38%	71th
5	Hou, Chih-yao	31	601	1.58	23	100%	76th
6	Duarte, Juan	23	925	1.77	51	79.55%	77th
7	Joe, Bina	23	746	1.8	32	58.33%	52th
8	Pepine, Carl J.	23	2450	2.41	69	82.04%	71th
9	Richards, Elaine M.	22	1165	5.32	22	91.89%	72th
10	Li, Jing	21	998	1.37	21	78.26%	55th

### 3.4. Analysis of journals

**Figure 5:**
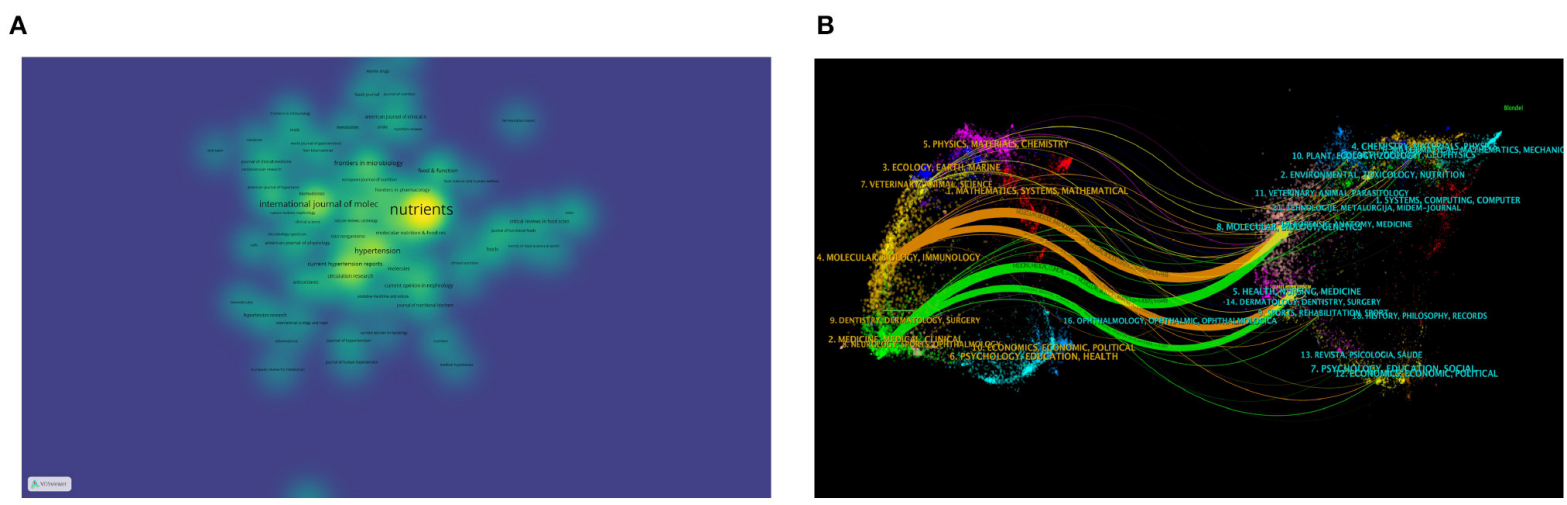
**(A)** Density map of journal article distribution using VOSviewer. **(B)** Dual-map overlay of journals in CiteSpace: citing journals are on the left, cited journals are on the right, colored paths indicate citation relationships.

Analyzing various journals can aid researchers in identifying suitable journals for submitting their articles. Table 3 displays the top fifteen journals based on publication volume. These journals, categorized as Q1 and Q2 (referring to the top 25 and 25–50% of journals in a specific field, respectively, based on their Impact Factor rankings), primarily concentrate on nutrition, cardiovascular, and molecular biology. Researchers in these areas may prioritize these journals for their submissions. Journals centered on nutrition feature the highest number of articles in this field. Among those related to cardiovascular studies, Circulation Research stands out with the highest Journal Impact Factor of 20.1. Notably, we observed that the majority of the top 15 Q1 journals are not open access (OA), a factor that could potentially impede scientific advancement in this field. Figure 5A presents a density plot depicting the number of articles indexed by journals, where brighter colors signify a higher count of indexed articles. Figure 5B offers a dual overlay of journals, illustrating the interconnectedness between various disciplines. For instance, clinical medicine is connected to molecular biology and nursing via green lines, signifying a close relationship among them.

**Table 3:** Top 15 journals with the highest number of publications.

**Rank**	**Source**	**Documents**	**JIF (2022)**	**JCI (2022)**	**JIF QUARTILE**	**Open access (OA)**
1	Nutrients	105	5.9	1.04	Q1	97.09%
2	International Journal of Molecular Sciences	47	5.6	0.71	Q1	97.88%
3	Hypertension	38	8.3	1.64	Q1	6.47%
4	Plos One	30	3.7	0.91	Q2	93.67%
5	Frontiers in Nutrition	27	5.0	0.90	Q2	94.44%
6	Frontiers in Microbiology	26	5.2	0.96	Q2	94.87%
7	Scientific Reports	26	4.6	1.06	Q2	95.03%
8	Current Hypertension Reports	23	5.6	0.71	Q2	17.59%
9	Frontiers in Physiology	23	4.0	1.00	Q2	92.48%
10	Food & Function	22	6.1	1.16	Q1	6.83%
11	Current Opinion in Nephrology and Hypertension	19	3.2	0.59	Q2	6.07%
12	Pharmacological Research	18	9.3	1.91	Q1	11.98%
13	Circulation Research	17	20.1	3.93	Q1	3.97%
14	Frontiers in Cellular and Infection Microbiology	17	5.7	0.83	Q1(Microbiology)	93.90%
15	Molecular Nutrition & Food Research	17	5.2	1.12	Q1	19.79%

### 3.5. Keyword analysis

**Figure 6:**
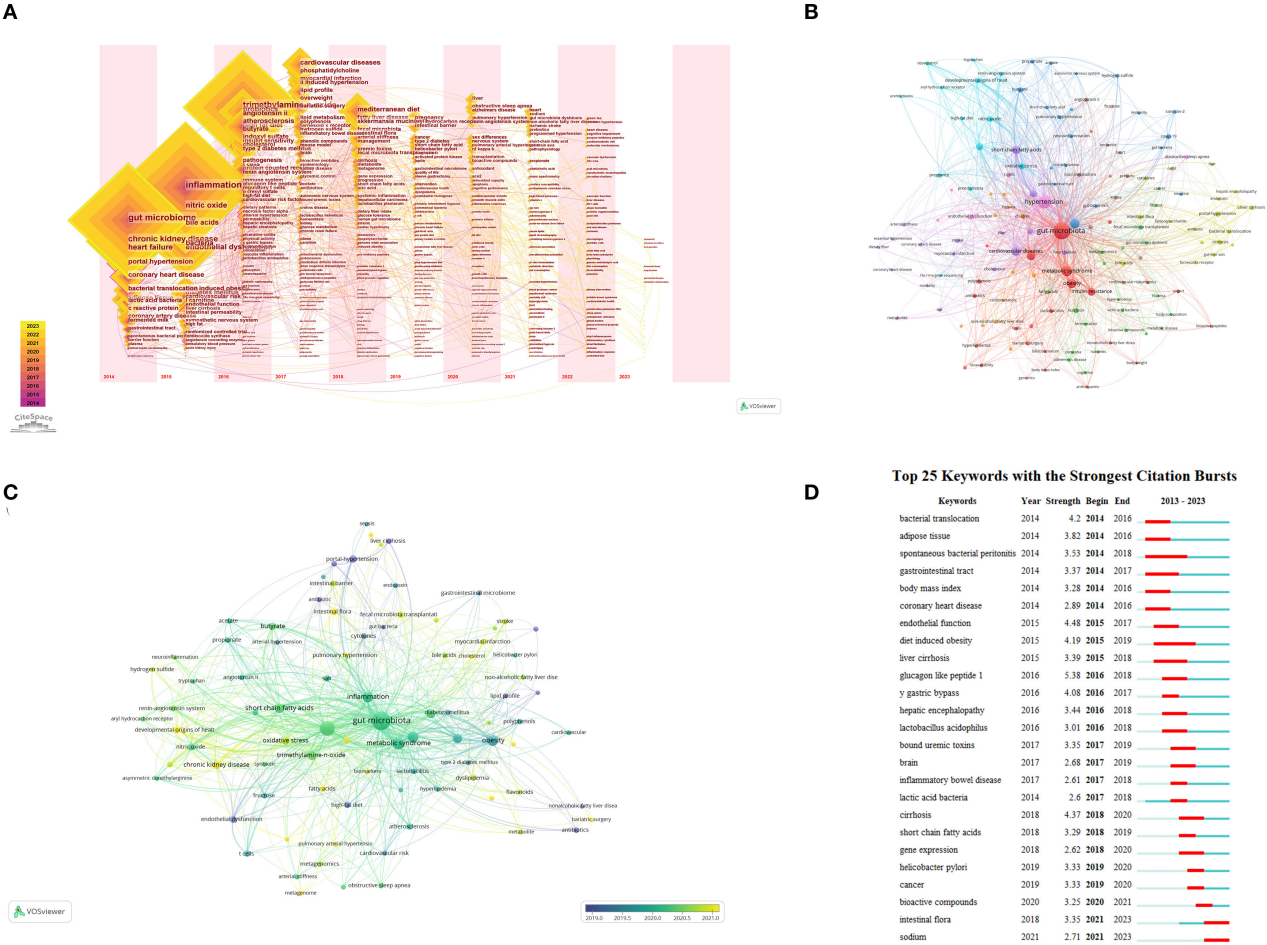
**(A)** Keyword timezone visualization from 2014 to 2023 using CiteSpace. **(B)** Network map of keywords using VOSviewer. **(C)** Timeline graphs of keywords using VOSviewer. **(D)** Top 25 keywords with the strongest citation bursts identified by CiteSpace.

Keyword analysis provides insights into the trending topics and emerging research directions in the study of GM and HTN (Table 4). Figure 6A showcases the top 15 keywords for both articles and review articles. To further understand these trends, we used CiteSpace and VOSviewer to perform a visual analysis of the keywords in this field. Figure 6A illustrates a CiteSpace timeline visualization of the keywords, demonstrating the temporal emergence of different keywords in the field. Prior to 2014, research related to GM was in progress but not deemed as a hot topic. In 2014, the field saw the emergence of keywords like hypertension, chronic kidney disease, bioactive peptides (fermented milk), and portal hypertension. The field gained substantial popularity from 2016 onwards, attracting a significant number of researchers. Around this time, prominent keywords included trimethylamine-N-oxide (TMAO), SCFAs, renin-angiotensin system, high-salt diet, and T cells. By 2019, new research hotspots surfaced with keywords such as fatty liver, tumors, gut-liver axis, myocardial infarction, and diabetes. In 2021 and 2022, this field became a major research hotspot. Alongside the aforementioned focus areas, there was a marked rise in articles related to sleep apnea-related hypertension, pulmonary arterial hypertension, gender differences, developmental origins of health and disease (DOHaD), GM and other cardiovascular diseases, and GM and other systemic diseases. Figure 6B presents the VOSviewer clustering results of the keywords, with GM and HTN serving as the central themes. Different-colored clusters represent different research directions in the field. In the red cluster, keywords such as metabolic syndrome, obesity, insulin resistance, anthocyanins, and Lactobacillus are closely linked. In the light blue cluster, HTN is closely associated with DOHaD, renin-angiotensin system, chronic kidney disease, and nitric oxide. In the dark blue cluster, short-chain fatty acids, TMAO, pulmonary arterial hypertension, central nervous system inflammation, and novel coronavirus are interconnected. The yellow cluster focuses on important research topics in the field of GM and HTN, such as SCFAs, gut-liver axis, portal hypertension, non-alcoholic fatty liver, and others. Figure 6C depicts the average time of different research directions in the field, with most research directions indicated by light green, suggesting that a majority of studies were conducted around 2020. This finding aligns with the increased publication volume of core authors previously discussed. Figure 6D displays the keyword burst analysis, using CiteSpace to identify keywords that have seen sudden growth within a certain period, indicative of emerging hotspots in the field. Glucagon-like peptide-1 is the keyword with the highest surge in frequency, while spontaneous bacterial peritonitis and obesity are the keywords with the longest burst duration. High salt, bioactive peptides, tumors, and other keywords are the newly emerging burst keywords in the field of GM and HTN.

**Table 4:** Top 15 keywords associated with the highest number of publications.

**Rank**	**Keywords (article)**	**Count**	**Centrality**	**Rank**	**Keywords (review article)**	**Count**	**Centrality**
1	Gut microbiota	717	0.12	1	Gut microbiota	438	0.06
2	Blood pressure	294	0.05	2	Blood pressure	240	0.15
3	Hypertension	171	0.05	3	Short-chain fatty acids	123	0.02
4	Metabolic syndrome	164	0.12	4	Cardiovascular disease	114	0.08
5	Obesity	131	0.02	5	Metabolic syndrome	103	0.02
6	Inflammation	108	0.02	6	Trimethylamine n-oxide	99	0.03
7	Short-chain fatty acids	100	0.07	7	Oxidative stress	90	0.02
8	Oxidative stress	88	0.21	8	Insulin resistance	76	0.02
9	Insulin resistance	79	0.06	9	Chronic kidney disease	60	0.06
10	Cardiovascular disease	68	0.04	10	Inflammation	60	0.03
11	Trimethylamine n-oxide	49	0.03	11	Hypertension	56	0.08
12	Chronic kidney disease	45	0.18	12	Heart failure	53	0.14
13	Nitric oxide	44	0.02	13	Bile acids	38	0.07
14	Endothelial dysfunction	39	0.05	14	Endothelial dysfunction	36	0.03
15	Probiotics	34	0.01	15	Cardiovascular diseases	34	0.02

### 3.6. Analysis of references

**Figure 7:**
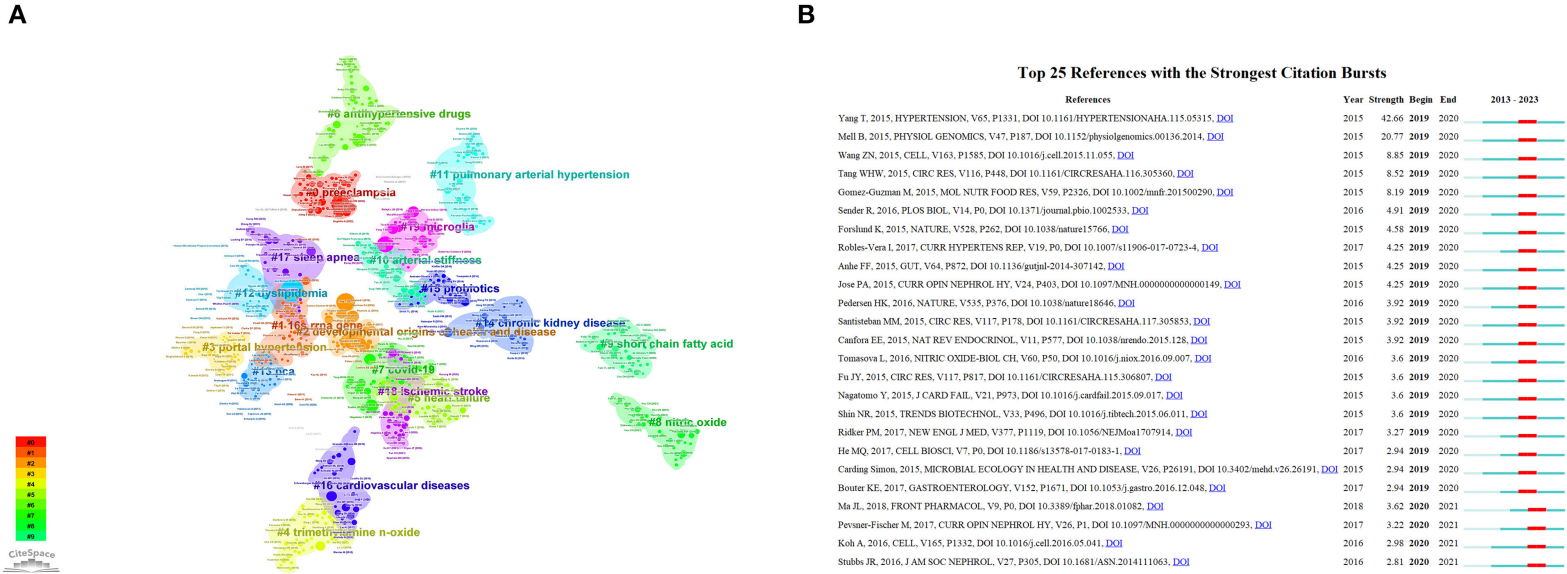
**(A)** The analysis of keyword clusters using citeSpace. **(B)** Top 25 references with the strongest citation bursts identified by citeSpace (2019–2023).

Analyzing references, which serve as a reservoir of knowledge, can provide insights into the foundational research within a specific field. Table 5 comprises the top 20 co-occurring references with a Category Normalized Citation Impact (CNCI) greater than four. Moreover, we examined the 30 most frequently cited references and identified distinctive research directions. Among these 30 articles, the bulk of them explored the influence of GM and its metabolites on HTN, thereby establishing a strong foundation for further investigations into the mechanisms of gut microbiota's role in HTN. Works by authors such as Yang Tao and Li Jing have garnered considerable attention. The remaining articles primarily delved into the mechanisms of action of GM and its metabolites, focusing on the roles of receptors like Olfr78 and Gpr41 in the function of short-chain fatty acids, and the interplay between GM and immune regulation influencing the cardiovascular system. We also conducted a clustering analysis (Figure 7A) on the references to facilitate the identification of the theoretical basis for various research directions. For instance, in the 2nd cluster, articles by Yang T (https://doi.org/10.1161/HYPERTENSIONAHA.115.05315), Adnan S (https://doi.org/10.1152/physiolgenomics.00081.2016), and Sun S (https://doi.org/10.1161/HYPERTENSIONAHA.118.12109) demonstrated that dysbiosis of gut microbiota can lead to increased blood pressure. This provides a foundation for the DOHaD study on how interventions on maternal GM can impact blood pressure in offspring rats. In the 11th cluster, articles by Kim S (https://doi.org/10.1161/HYPERTENSIONAHA.119.14294), Sharma RK (https://doi.org/10.1161/CIRCRESAHA.118.313882), and Yang T (https://doi.org/10.3389/fphys.2017.00845) discussed the role of the immune system in HTN, the unique gut microbiota in pulmonary arterial hypertension, and so on, laying the groundwork for exploring the mechanisms of gut microbiota in the onset and progression of pulmonary arterial hypertension. Finally, we performed a burst analysis (Figure 7B) on the references from 2019 to 2023 to identify the articles that suddenly gained popularity in this field. As depicted in the figure, the most cited references in the past five years were primarily from 2015 to 2017. Furthermore, the field received significant attention from 2019 to 2021. The article by Yang Tao presented the highest burst intensity of 42.66. In 2021, four highly regarded review articles (the last four articles) predominantly discussed the metabolism of microbiota metabolites and the interaction mechanisms between microbiota and cardiovascular diseases.

**Table 5:** Top 20 References with the highest number of citations.

**Rank**	**Cited references**	**Count**	**Centrality**	**CNCI**
1	Li J, 2017, MICROBIOME, V5, PO, DOI 10.1186/s40168-016-0222-x	326	0.02	13.83
2	Yang T, 2015, HYPERTENSION, V65, Pl331, DOI 10.1161/HYPERTENSIONAHA.115.05315	218	0.05	14.09
3	Marques FZ, 2017, CIRCULATION, V135, P964, DOI 10.1161/CIRCULATIONAHA.116.024545	206	0.00	8.57
4	Santisteban MM, 2017, CIRC RES, V120, P312, DOI 10.1161/CIRCRESAHA.116.309006	171	0.04	4.94
5	Wilck N, 2017, NATURE, V551, P585, DOI 10.1038/nature24628	153	0.01	10.99
6	Kim S, 2018, CLIN SCI, V132, P701, DOI 10.1042/CS20180087	140	0.03	7.17
7	Mell B, 2015, PHYSIOL GENOMICS, V47, P187, DOI 10.1152/physiolgenomics.00136.2014	115	0.09	4.26
8	Marques FZ, 2018, NAT REV CARDIOL, V15, P20, DOI 10.1038/nrcardio.2017.120	109	0.08	5.99
9	Bartolomaeus H, 2019, CIRCULATION, V139, P1407, DOI 10.1161/CIRCULATIONAHA.118.036652	106	0.18	8.36
10	Tang WHW, 2017, CIRC RES, V120, P1183, DOI 10.1161/CIRCRESAHA.117.309715	105	0.00	15.12
11	Natarajan N, 2016, PHYSIOL GENOMICS, V48, P826, DOI 10.1152/physiolgenomics.00089.2016	91	0.00	4.41
12	Yang T, 2018, NAT REV NEPHROL, V14, P442, DOI 10.1038/s41581-018-0018-2	80	0.00	9.00
13	Jie ZY, 2017, NAT COMMUN, V8, P0, DOI 10.1038/s41467-017-00900-1	80	0.02	13.44
14	Zhu WF, 2016, CELL, V165, P111, DOI 10.1016/j.cell.2016.02.011	71	0.05	20.46
15	Khalesi S, 2014, HYPERTENSION, V64, P897, DOI 10.1161/HYPERTENSIONAHA.114.03469	58	0.05	6.38
16	Tang WHW, 2015, CIRC RES, V116, P448, DOI 10.1161/CIRCRESAHA.116.305360	55	0.25	11.74
17	Koh A, 2016, CELL, V165, P1332, DOI 10.1016/j.cell.2016.05.041	43	0.00	25.00
18	Wang ZN, 2015, CELL, V163, P1585, DOI 10.1016/j.cell.2015.11.055	41	0.03	12.85
19	Schiattarella GG, 2017, EUR HEART J, V38, P2948, DOI 10.1093/eurheartj/ehx342	35	0.04	5.70
20	Chen ML, 2016, MBIO, V7, P0, DOI 10.1128/mBio.02210-15	34	0.02	8.66

## 4. Discussion

### 4.1. General information

The field of GM and HTN has seen contributions from a total of 88 countries/regions and 9,573 authors, resulting in 1,730 articles. These articles were spread across 593 journals, with 1,000 references co-occurring more than 10 times. The volume of relevant studies in the area of GM and HTN has been on an upward trajectory in recent years, marking it as a burgeoning research hotspot. We anticipate that this field will continue to garner increased attention moving forward. While China leads in the number of publications, the average citation per article is relatively low. The United States, although second in terms of publication volume, holds a central position in the research field, indicated by its extensive collaborations with other countries. Its most frequent collaborator is China. Our analysis of authors extends beyond merely considering the number of publications and H-index. It's evident that researchers from European and American countries have a longer history of studying this field, while Chinese researchers entered the field more recently. Furthermore, stable research communities have formed within the field, with individuals like Tain You-Lin, Hsu Chien-Ning, and Chih-Yao Hou holding pivotal roles in the field of developmental origins of health and disease. Journals in the domains of nutrition, cardiovascular research, and molecular biology have published the greatest number of articles in this field. Researchers looking to make manuscript submissions in this area should prioritize these journals.

### 4.2. Hot-spots and frontiers

Bibliometrics serve a crucial role in organizing key trends and advancing frontiers within a field. In our study, we employed tools such as CiteSpace and VOSviewer to analyze author keywords and references in this field. The objective was to identify seminal and innovative articles, and to objectively encapsulate different research directions. The interplay between GM and HTN has remained a solid research focal point from 2015 to 2022, with a large contingent of scholars participating actively, particularly between 2018 and 2021. This period saw considerable contributions that enriched the exploration within this area. The correlation between GM and heart failure has recently come into focus, surfacing around 2017, and gaining momentum with 14 publications in 2021 and rising to 28 in 2022. Investigations into the relationship between GM and coronary heart disease commenced around 2015, and those concerning GM and myocardial infarction started around 2017, with an average of 7–8 publications per annum. However, the linkage between GM and other cardiovascular diseases, such as hypertrophic cardiomyopathy, endocarditis, and arteritis, is less represented in the literature. This suggests that these diseases could become new research hotspots in the future. The association of GM with maladies such as metabolic syndrome, chronic kidney disease, pulmonary arterial hypertension, portal hypertension, and liver cirrhosis has also been a strong research hotspot, with a wealth of articles available on these subjects. The research is complex given GM's involvement in the progression of a multitude of diseases. Consequently, our analysis concentrated primarily on the significant clusters directly related to HTN.

#### 4.2.1. GM and their mechanisms of action

Prior to 2014, research on the connection between GM and HTN was scant. A handful of clinical trials hinted that dietary modifications could improve weight, insulin sensitivity, blood lipids, and blood pressure in obese patients, coinciding with shifts in GM (11). Yet, a direct causal link between GM and HTN remained unproven, prompting some researchers to suggest GM as a potential therapeutic avenue for HTN. In 2015, a breakthrough came when Yang et al. (3) team conducted a gut genomic analysis on a select group of hypertensive patients and various hypertensive rat models. The findings illustrated comparable disruptions in gut bacteria proportions and SCFAs in both hypertensive humans and rats, signifying a strong correlation between GM dysbiosis and high blood pressure (3). Fast forward to 2017, Li et al. analyzed the metagenomic composition of fecal samples from healthy individuals, prehypertensive subjects, and primary hypertensive patients (4). They discovered that the microbial species in prehypertensive and hypertensive individuals closely resembled each other and significantly differed from those in healthy individuals, further reinforcing the link between microbiota dysbiosis and HTN. Moreover, the team transplanted fecal samples from hypertensive patients into germ-free mice with normal blood pressure. This transplantation led to an increase in the mice's blood pressure, furnishing direct evidence of GM dysbiosis's role in HTN (4). In 2020, Joonatan Palmu and his team conducted a genomic analysis of GM in 6,953 Finnish individuals, unearthing significant species variations such as lactobacilli, which displayed a negative correlation with HTN (12). Another study involving 4,672 participants from six different ethnic backgrounds underscored not only a connection between GM composition and blood pressure but also notable differences in microbiota composition among diverse ethnic groups (13). These investigations robustly endorse GM dysbiosis as a contributing factor in HTN, paving the way for further explorations into the link between GM and cardiovascular diseases. Works from researchers such as Yang Tao and Li Jing continue to garner considerable attention within the scientific community, ranking within the top 1% in the field of clinical medicine based on their high citation thresholds relative to their respective field and year of publication.

Once the link between GM and HTN was established, the mechanisms by which GM influences HTN piqued the interest of a multitude of researchers, making this a hotbed of scientific exploration. In 2017, more than 110 papers were published in this field, reflecting an annual growth of approximately 80 papers. A central focus of recent research is the influence of abnormal GM metabolites on blood pressure. The interest is particularly piqued by metabolites such as SCFAs, trimethylamine N-oxide (TMAO), and secondary bile acids. SCFAs are generally perceived as beneficial to human health. Research has revealed that hypertensive patients exhibit decreased levels of SCFAs (acetate and butyrate) in their blood, correlating with elevated blood pressure (3, 14). Supplementation with acetate (dietary fiber) in hypertensive rats resulted in significant reductions in systolic and diastolic blood pressures and mitigated fibrosis in the heart and kidneys (5). Moreover, supplementing SCFAs to high-fat diet-fed pregnant mice led to markedly lower blood pressure in their male offspring (15). SCFAs exert their antihypertensive effects primarily through immune regulation or receptor binding. They are known to modulate T cells and exhibit anti-inflammatory properties (16, 17), with their anti-inflammatory mechanism mediated by regulating the NLRP3 inflammasome (18). Two known receptors for SCFAs, Olfr78, and G-protein-coupled receptor 41 (Gpr41), are implicated in blood pressure reduction when bound to SCFAs (19). In particular, Gpr41 can regulate blood pressure by reducing arterial vascular tone (20). Recently, additional short-chain fatty acid receptors were discovered, including Gpr43, Gpr109A, and Olfr558 (14, 21). In contrast, TMAO is generally regarded as harmful to the body. A cohort study involving 4,007 participants found an association between increased plasma levels of TMAO and the incidence of cardiovascular events (22). TMAO is known to elevate the risk of cardiovascular disease via mechanisms such as Ang-II activation of the MAPK pathway, increased platelet reactivity, and inflammation promotion (23–25). Hence, TMAO inhibition can enhance cardiovascular disease prognosis (26–28). Notably, TMAO has become a prognostic indicator for various cardiovascular diseases, including atherosclerosis, heart failure, and pulmonary arterial hypertension (29–32). However, some studies have indicated that excessive SCFAs can induce Th1 and Th17 cell proliferation, which promotes inflammation (33), while some TMAO precursors have been found to exert protective effects on the cardiovascular system (34). Research has also shown that hypertensive rats exhibit impaired intestinal barrier function, increased inflammation, and enhanced sympathetic nerve impulses in the gut. However, supplementation with SCFAs lowers blood pressure in rats and improves intestinal barrier function (35, 36). Despite these findings, a causal relationship between the intestinal barrier, GM, and HTN has not been definitively established. Lastly, the influence of GM on the metabolism of antihypertensive drugs, which consequently affects the efficacy of HTN treatment, has also been a focal point of research.

Beyond the mechanisms through which GM impacts HTN, the association between GM and other diseases such as coronary heart disease, myocardial infarction, heart failure, and endothelial dysfunction (represented by the purple cluster in Figure 6B) is gradually emerging as a key research area for numerous scholars.

#### 4.2.2. Salt-sensitive hypertension

Salt-sensitive hypertension (SSH) has emerged as a significant area in the field of GM and HTN research. As depicted in Figure 6C, the average research period for SSH in this field is around 2020, closely tied to keywords such as inflammation, renin-angiotensin system, SCFAs, and metabolic syndrome. In 2015, Blair Mell and colleagues built upon prior research, discovering that Dahl rats on a high-salt diet displayed increased blood pressure and altered GM. Notably, there was an increase in the S24-7 family of the Bacteroidetes phylum and the Veillonellaceae family of the Firmicutes phylum (37). Despite these findings, the mechanisms through which GM influences SSH remained unclear until around 2017 when explorations into the relevant mechanisms began. In 2018, a study by Bier, A et al. examined the SCFAs in the feces of Dahl rats on a high-salt diet and observed a significant decrease in acetate compared to the control group (38). This finding suggests that high salt intake might influence salt-sensitive blood pressure through its effect on SCFAs. Concurrently, a cohort study of 145 individuals found that lowering sodium intake in hypertensive patients led to increased circulating SCFAs and decreased blood pressure (16). High salt intake has been shown to raise blood pressure by triggering inflammation (39–41). Several studies have highlighted the role of GM in inflammation induced by high salt intake: (1) Rats on a long-term high-salt diet showed elevated levels of TMAO in the systemic circulation and brain. TMAO is known to increase blood pressure by influencing central cardiovascular regulatory centers, promoting neural inflammation, and inducing oxidative stress (42). (2) Another critical study showed that a high-salt diet not only elevated inflammation-associated Th17 cells but also resulted in a decrease in gut lactate sensing (43). These studies set the stage for further research into the potential modulation of GM to alleviate inflammatory cell infiltrations and reduce blood pressure. Over the past three years, recent research has indicated that a high-salt diet can also affect HTN by altering the metabolism of amino acids by the GM (44, 45). For instance, a high-salt diet can modify GABA and glutamate/glutamine metabolism as well as glycolysis-related amino acid metabolism, thereby exacerbating HTN.

#### 4.2.3. Developmental origins of health and disease

The developmental origins of health and disease (DOHaD) concept pertains to the long-lasting effects of certain environmental influences on the structure or function of organisms during early life (15). For instance, an improper diet during pregnancy can lead to various pathological alterations in male offspring, including HTN. However, timely intervention during pregnancy can mitigate high blood pressure in these offspring. In Figure 6B, DOHaD is depicted by a light blue cluster and closely connected with keywords such as SCFAs, TMAO, oxidative stress, chronic kidney disease, and the renin-angiotensin system with an average research year of 2021 (Figure 6C). Before 2014, a handful of studies had noted changes in the GM of pregnant women under the influence of external factors (46). In 2015, Tain et al. made the discovery that high salt intake can induce HTN in the offspring of mice fed a high-fructose diet under the DOHaD concept (47). Around 2017, researchers such as Tain, You-Lin, Hsu, Chien-Ning, and Chih-Yao Hou from the Kaohsiung Chang Gung Memorial Hospital established a strong link between DOHaD and GM. By delving into the underlying mechanisms, they published over 40 articles within this field, solidifying their positions as leading figures. For instance, they found that high-fat, high-fructose, and tryptophan-free diets in pregnant mice led to increased blood pressure in male offspring. However, when the diets of these pregnant mice were supplemented with SCFAs, antibiotics, and butyrate, a significant decrease in the blood pressure of their male offspring was observed, compared to the control group (15, 48, 49). By 2020, mechanisms explored in this field included the renin-angiotensin system, nitric oxide, hydrogen sulfide, oxidative stress, and microbial metabolites. Furthermore, chronic kidney disease has also emerged as a significant research direction within the intersection of DOHaD and GM.

#### 4.2.4. Obstructive sleep apnea-induced hypertension

Obstructive sleep apnea (OSA) is a sleep-related respiratory disorder defined by symptoms such as snoring, respiratory pauses, and excessive daytime sleepiness. The research linking OSA and GM has predominantly revolved around keywords like T cells, HTN, and SCFAs, particularly around May 2020 (Figure 6B, pink cluster). Over the last two decades, multiple clinical trials have established a causal link between OSA and HTN (50). In 2016, a study led by Durgan et al. utilized rats to model OSA, feeding them either a high-fat diet or a regular diet (51). Their findings revealed that rats on the high-fat diet exhibited increased blood pressure and significant alterations in GM, while the rats on the regular diet maintained normal blood pressure. Interestingly, when GM from rats on the high-fat diet was transplanted into rats with normal blood pressure, the recipient rats experienced an increase in blood pressure (51). This experiment signified a causal link between GM and HTN in OSA, offering valuable direction for future research in this area. In 2018, researchers Ganesh and Durgan implemented an intervention using SCFAs in OSA rats. They found that suitable increases in cecal SCFAs concentration could prevent OSA-induced intestinal inflammation and HTN (52). More recently, a clinical study has indicated that OSA patients not only display an imbalance in GM but also an altered Th17/Treg cell ratio (53).

#### 4.2.5. Antihypertensive peptides

Antihypertensive Peptides (ACEIPs) are bioactive peptides generated through fermentation by specific GM, exhibiting antioxidant, anti-inflammatory, and blood pressure-lowering properties (54). ACEIPs achieve their antihypertensive effects through the inhibition of the angiotensin-converting enzyme and the stimulation of angiotensin-converting enzyme 2 pathways (55, 56). As shown in Figure 6B, ACEIP, depicted by the red cluster, is closely tied to keywords like HTN, metabolic syndrome, and insulin resistance. Around 2014, comprehensive research on ACEIP revealed its beneficial effects on metabolic syndrome, including blood pressure reduction and improved insulin resistance. These benefits, derived from ACEIP extracted from animal sources, fermented milk, and other sources, have been confirmed through clinical trials. Presently, researchers are primarily interested in investigating the roles of various types of ACEIP in reducing blood pressure. For instance, numerous ACEIP with impacts on cardiovascular disease have been extracted from plants such as Rumex vesicarius, navy bean, and hidakakombu (57–59). Additionally, ACEIP extracted from human breast milk, cow milk, camel milk, and other animal sources have been examined (60–62), with over 100 ACEIP extracted from dairy products alone (63). Due to the incorporation of both animal and plant studies, some articles within this field have found their place in agriculture-related journals.

## 5. Conclusion

The study of GM and HTN has become an increasingly prominent research area, drawing the attention of a rising number of researchers. Numerous experiments have established a causal relationship between GM and HTN. However, the interaction mechanisms between GM and HTN still hold many uncertainties, necessitating further investigation. Concurrently, GM may indirectly influence the development of HTN by affecting the progression of specific diseases. Additionally, the impact of GM on other cardiovascular diseases is progressively garnering interest among researchers. To summarize, we employed bibliometric tools to analyze all articles within this field over the past decade, highlighting important and innovative works across various research directions, uncovering hotspots and emerging trends, and offering valuable insights for future research endeavors.

## Author contributions

QJ: Supervision, Writing—review and editing. YJ: Conceptualization, Methodology, Writing—original draft, Writing—review and editing. WL: Data curation, Methodology, Software, Visualization, Writing—original draft. QZ: Data curation, Software, Visualization, Writing—review and editing.
